# Substrate-Specific Reorganization of the Conformational Ensemble of CSK Implicates Novel Modes of Kinase Function

**DOI:** 10.1371/journal.pcbi.1002695

**Published:** 2012-09-20

**Authors:** Michael A. Jamros, Leandro C. Oliveira, Paul C. Whitford, José N. Onuchic, Joseph A. Adams, Patricia A. Jennings

**Affiliations:** 1 Department of Chemistry and Biochemistry, University of California San Diego, La Jolla, California, United States of America; 2 Laboratório Nacional de Ciência e Tecnologia do Bioetanol – CTBE/CNPEM, Campinas, São Paulo, Brazil; 3 Center for Theoretical Biological Physics, Rice University, Houston, Texas, United States of America; 4 Department of Pharmacology, University of California San Diego, La Jolla, California, United States of America; National Cancer Institute, United States of America and Tel Aviv University, Israel

## Abstract

Protein kinases use ATP as a phosphoryl donor for the posttranslational modification of signaling targets. It is generally thought that the binding of this nucleotide induces conformational changes leading to closed, more compact forms of the kinase domain that ideally orient active-site residues for efficient catalysis. The kinase domain is oftentimes flanked by additional ligand binding domains that up- or down-regulate catalytic function. C-terminal Src kinase (Csk) is a multidomain tyrosine kinase that is up-regulated by N-terminal SH2 and SH3 domains. Although the X-ray structure of Csk suggests the enzyme is compact, X-ray scattering studies indicate that the enzyme possesses both compact and open conformational forms in solution. Here, we investigated whether interactions with the ATP analog AMP-PNP and ADP can shift the conformational ensemble of Csk in solution using a combination of small angle x-ray scattering and molecular dynamics simulations. We find that binding of AMP-PNP shifts the ensemble towards more extended rather than more compact conformations. Binding of ADP further shifts the ensemble towards extended conformations, including highly extended conformations not adopted by the apo protein, nor by the AMP-PNP bound protein. These ensembles indicate that any compaction of the kinase domain induced by nucleotide binding does not extend to the overall multi-domain architecture. Instead, assembly of an ATP-bound kinase domain generates further extended forms of Csk that may have relevance for kinase scaffolding and Src regulation in the cell.

## Author Summary

The Src protein kinases are integral members of numerous signaling pathways involved in cellular growth and differentiation. The master regulator of the Src family is the protein kinase Csk, which adds a phosphate to the C-terminal tail, inhibiting Src Kinase function. Proper regulation of these signaling pathways by Csk is essential as unregulated activity in these pathways is correlated with the development of various cancers and autoimmune diseases. Understanding the nature of the mechanism and structure of Csk may lead to therapeutics and a better understanding of Src signaling pathways. Conformational changes associated with nucleotide binding and release have been shown to regulate the efficiency of Src down-regulation by Csk. To obtain insights into the nature of these nucleotide-induced structural changes, we examined the conformation of Csk in solution while bound to the ATP analog AMP-PNP and product ADP using a combination of small angle x-ray scattering and molecular dynamics. Surprisingly, both nucleotides induce extended conformations of Csk compared to the apo-enzyme, suggesting a novel mode of function. Further understanding of this mode of function may aid in the design of cancer therapeutics that act by regulating Src signaling pathways by modulating the function of Csk.

## Introduction

The Src family of tyrosine kinases (SFKs) is comprised of modular signaling enzymes involved in the control of cellular growth and differentiation. The members of this family contain three important structural domains: a C-terminal tyrosine kinase domain (comprised of a small and large lobe), which is preceded in sequence by the non-catalytic regulatory SH2 and SH3 domains [1], [2], [3], [4]. While phosphorylation of the activation loop is autocatalytic, phosphorylation of the C-terminal tail is inhibitory and requires Csk [5]. Csk contains the same structural domains as SFKs, but lacks an inhibitory C-terminal tail and an N-terminal sequence for membrane localization [6]. Additionally, Csk is not regulated through phosphorylation of its activation loop. Instead, Csk is constitutively active and increased activity is coupled to its association with membrane adaptor proteins.

Each domain of Csk plays a role in interacting with various binding partners. The kinase domain binds and phosphorylates all nine members of the Src family [7], a process that requires the binding of ATP and magnesium. The SH2 domain of Csk is responsible for binding a large number of scaffolding proteins. Since Csk is a cytosolic protein and the substrate Src is membrane localized, localization of Csk to Src requires interaction between the Csk SH2 domain and several scaffolding proteins. These scaffolding proteins include the ubiquitously expressed transmembrane protein Cbp, Caveolin-1, Paxillin, the insulin receptor substrate IRS-1, and Helicobacter pylori CagA [8], [9], [10], [11]. Lastly, the SH3 domain has been shown to bind PKA and the phosphatase PEP [12], [13].

The gamut of binding partners for Csk suggests it must be an adaptable protein in order to carry out all of its known functions. One aspect that may be central to this adaptability is nucleotide-derived conformational changes. Pre-steady-state kinetic studies suggest that a slow conformational change in Csk limits Src phosphorylation [14], [15]. In addition, previous work from our lab examined the effects of nucleotide binding on Csk using hydrogen-deuterium exchange mass spectrometry (DXMS) and found binding of the ATP analog AMP-PNP as well as ADP led to changes in the protection of multiple peptides [16], [17], [18]. These data suggest that nucleotide binding to Csk has conformational effects on the protein that extend beyond the nucleotide pocket in the kinase domain and into the SH2 domain, the site of adaptor protein binding and catalytic regulation. While these studies establish the existence of long-range communication across Csk, they do not provide a structural framework in which to understand these observed inter-domain relationships.

Small angle x-ray scattering (SAXS) and NMR studies combined with molecular dynamics simulations has become an increasingly powerful tool to understand the conformational ensemble properties of proteins in solution [19], [20], [21], [22], [23], [24], [25], [26]. Using a combination of SAXS and structure-based models (SBM), or SAXS-SBM, we showed that apo Csk is comprised of an ensemble of extended and compact conformations in solution, rather than adopting a single conformational form as depicted by the X-ray structure [27]. Here, we applied these methods to Csk to ask how nucleotide binding affects the conformational landscape of this flexible multi-domain kinase. We show that upon binding of the ATP analog AMP-PNP, there is a shift in the conformational ensemble of Csk towards more extended, open conformations. When bound to the reaction product ADP, the ensemble shifts even further towards these extended conformations. These data suggest that the orientation of regulatory domains in Csk is highly sensitive to the presence of nucleotides in the kinase domain. Most importantly, rather than inducing more compact forms, occupancy of the nucleotide pocket in the kinase domain induces more open forms of Csk.

## Results

### Solution Scattering of Csk

We previously collected SAXS data on the apo form of Csk and identified an ensemble of compact and extended conformations that describe the protein in solution [27]. In the current study, we asked whether or not the binding of nucleotides to Csk might alter this ensemble. DXMS studies comparing apo Csk to nucleotide bound Csk (AMP-PNP and ADP) displayed changes in protection of many peptides, which suggests both the binding of ATP and delivery of the γ phosphoryl group of ATP induce global conformational changes in Csk [16]. In order to obtain a structural description of how binding of these nucleotides alters the conformation of Csk, we collected SAXS data on AMP-PNP and ADP bound Csk.

**Figure 1:**
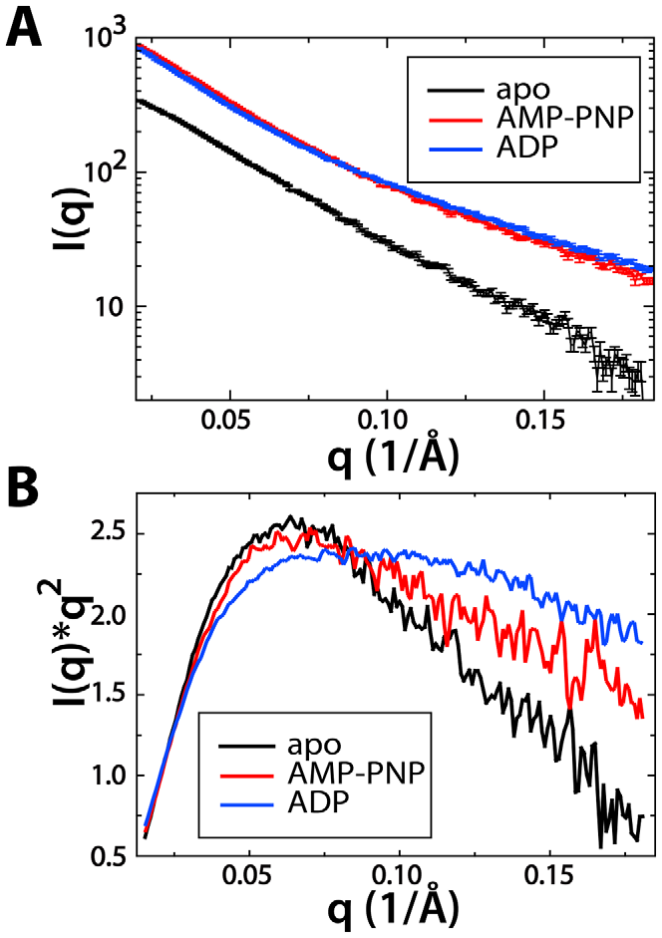
Small-angle X-ray scattering curves and Kratky plot of experimental data. A) Solution scattering data for apo Csk (black), AMP-PNP bound Csk (red), and ADP bound Csk (blue). B) Kratky plot of experimental solution scattering data colored in the same manner as A. Scattering data is normalized for ease of comparison in the plot.

To determine if there are significant global changes in the solution structure of these bound states, we initially compared the scattering profile of each protein using a Kratky plot (Figure 1). Kratky plots (I(q)*q^2^ versus q) provide a qualitative analysis of the “folded-ness” of a protein. Globular proteins display a bell shaped curve, whereas extended proteins lack a peak and will either plateau, or have elevated I(q)*q^2^ values for higher q values [28]. The apo protein plot indicates that Csk is well-folded with flexible linkers, which is consistent with our previous SAXS analysis, as well as crystallographic data [27], [29]. However, both the AMP-PNP and ADP bound protein show signs of possible disorder and local unfolding, as indicated by the higher I(*q*)*q^2^ values at larger scattering angles (higher *q*). While the AMP-PNP bound protein data shows moderate deviations from the apo, the ADP bound protein shows a dramatic shift towards more disorder. Additionally, the experimentally determined radius of gyration (R_g_) values show the apo protein (R_g_ = 39 Å) to be more compact than the AMP-PNP bound (R_g_ = 40 Å) and the ADP bound protein (R_g_ = 44 Å). Taken together, these data suggest significant shifts in the conformations of Csk when nucleotide is bound to the protein.

### Generation of Candidate Conformations via Molecular Dynamics

**Figure 2:**
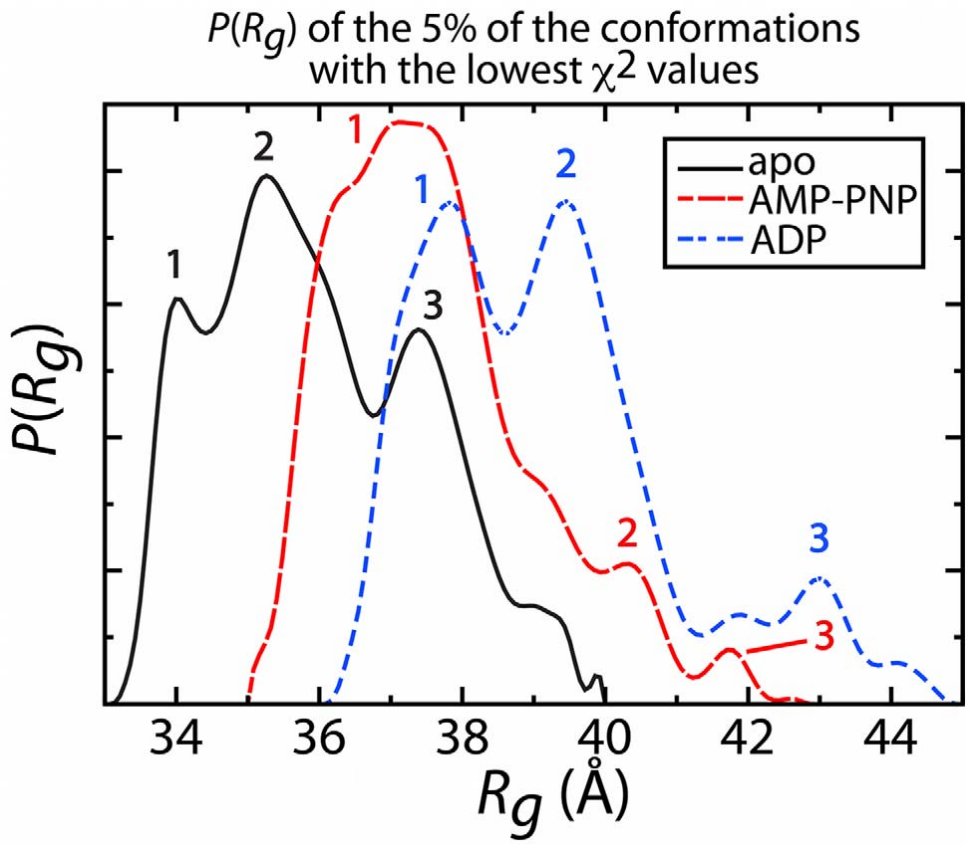
The probability of R_g_ (P(R_g_)) of the 5% of conformations with the lowest χ^2^ values from the SBM simulations. Apo Csk appears in black, AMP-PNP bound Csk appears in red, and ADP bound Csk appears in blue. The three most prominent populations, from which representative conformations were selected for ensemble analysis, are highlighted for each state.

To further investigate the nature of Csk's conformational change upon nucleotide binding, we modeled the experimental scattering data using SBM. In our previous studies using SAXS-SBM we showed the crystal structure does not accurately describe apo Csk in solution, but rather it is composed of an ensemble of compact and extended conformational states. The higher experimental R_g_ values of both the AMP-PNP and ADP bound forms of Csk suggest an ensemble of structures that are on average more extended than the apo protein. Using an all-atom SBM, we generated 40,000 candidate conformations for analysis. Theoretical scattering curves were generated for each individual conformation and then compared to the experimental scattering curve for all states of Csk using χ^2^ to measure the goodness of fit. Specifically, χ^2^ is a measure of the discrepancy between the theoretical and the experimental curves (see Methods for more detail). For each experimental curve we selected candidate conformations from the simulation, using the 5% of conformations with the lowest χ^2^ values for each case. We then calculated P(R_g_) (R_g_ values calculated using the g_gyrate module of Gromacs), the probability in R_g_ space, for all low-χ^2^ conformations (Figure 2). This process effectively applies a low-χ^2^ filter to the candidate conformations, which range from R_g_ = 25.5 Å to R_g_ = 45.1 Å. In addition, we generated P(R_g_) plots for the 2.5% and 7.5% of conformations with the lowest χ^2^ values and the data were consistent, ensuring the robustness of the 5% selection criteria.

**Figure 3:**
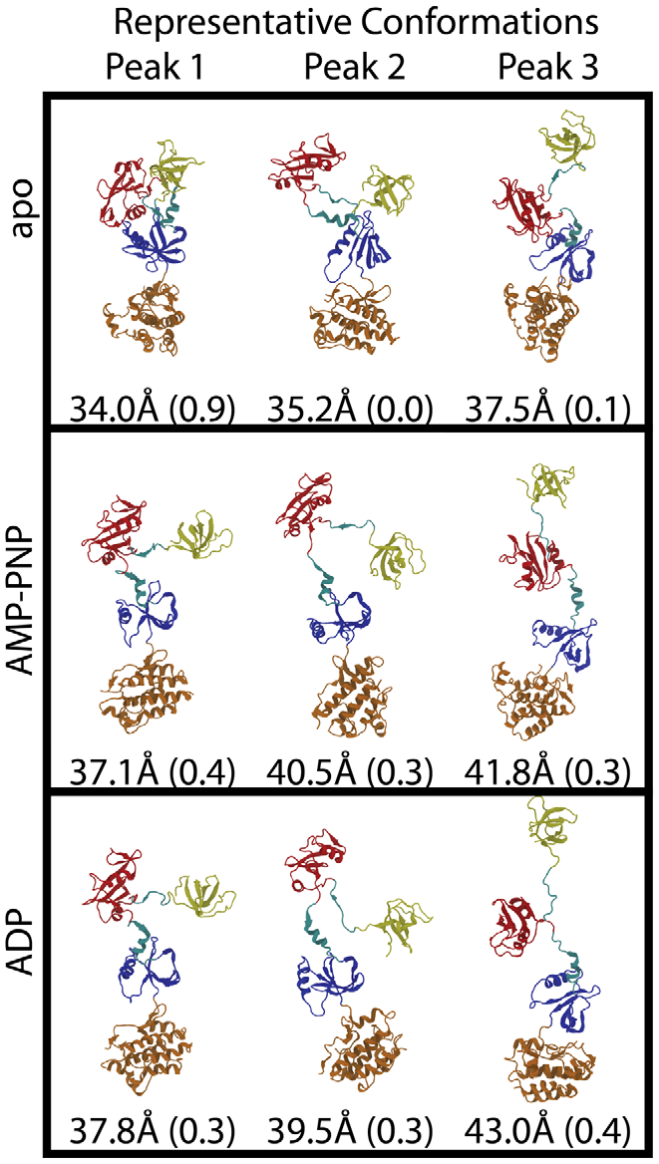
Representative conformations from the peaks of the P(R_g_) plot, along with their R_g_ values. Appearing next to the R_g_ values of each conformation is the fraction of the best-fit ensemble made up by that conformation. For all conformations the SH3 domain appears in yellow, the SH2 domain appears in red, the N-lobe of the kinase domain appears in blue, the C-lobe of the kinase domain appears in orange, and the domain-domain linkers appear in cyan.

The low χ^2^ filtered sets each possess multiple distinct peaks, representing likely conformational populations of Csk in solution. These populations most likely contribute to the experimental signal, but the ensemble distribution in not extracted from P(R_g_) directly (see Methods). The apo protein prefers the most compact conformations, with peaks at ∼34.0 Å, ∼35.2 Å, and ∼37.5 Å, which is consistent with our previous analysis [27]. The AMP-PNP protein displays a shift towards more extended conformations with one major peak at ∼37.8 Å and two minor peaks at ∼39.5 Å and ∼41.6 Å. Lastly, the ADP bound protein displays two major peaks at ∼37.1 Å and ∼40.5 Å, as well as a minor but potentially significant population at ∼43.0 Å. Representative conformations from all peaks are depicted in Figure 3.

Common to all states of Csk studied here, we observe a similar major population (R_g_ ∼37–38 Å) that fits the experimental data well. However, each state of Csk displays conformations of differing R_g_ values that also describe the experimental data. The apo protein adopts more compact conformations than those observed in either of the nucleotide bound states. Upon binding of AMP-PNP, the predominant population (peak 1) covers a broad R_g_ range centered around a peak at ∼37 Å, shifting away from the compact structures observed for the apo protein. When ADP is bound, the P(R_g_) shifts even further towards higher R_g_ values, displaying two populations at high R_g_ that are not among the low χ^2^ conformations for the apo and AMP-PNP bound protein. Overall, the apo protein is the most compact form, binding of AMP-PNP shifts towards the more extended structures, and binding of ADP shifts to populations of even more extended conformations.

### Defining the Conformational Ensemble

**Figure 4:**
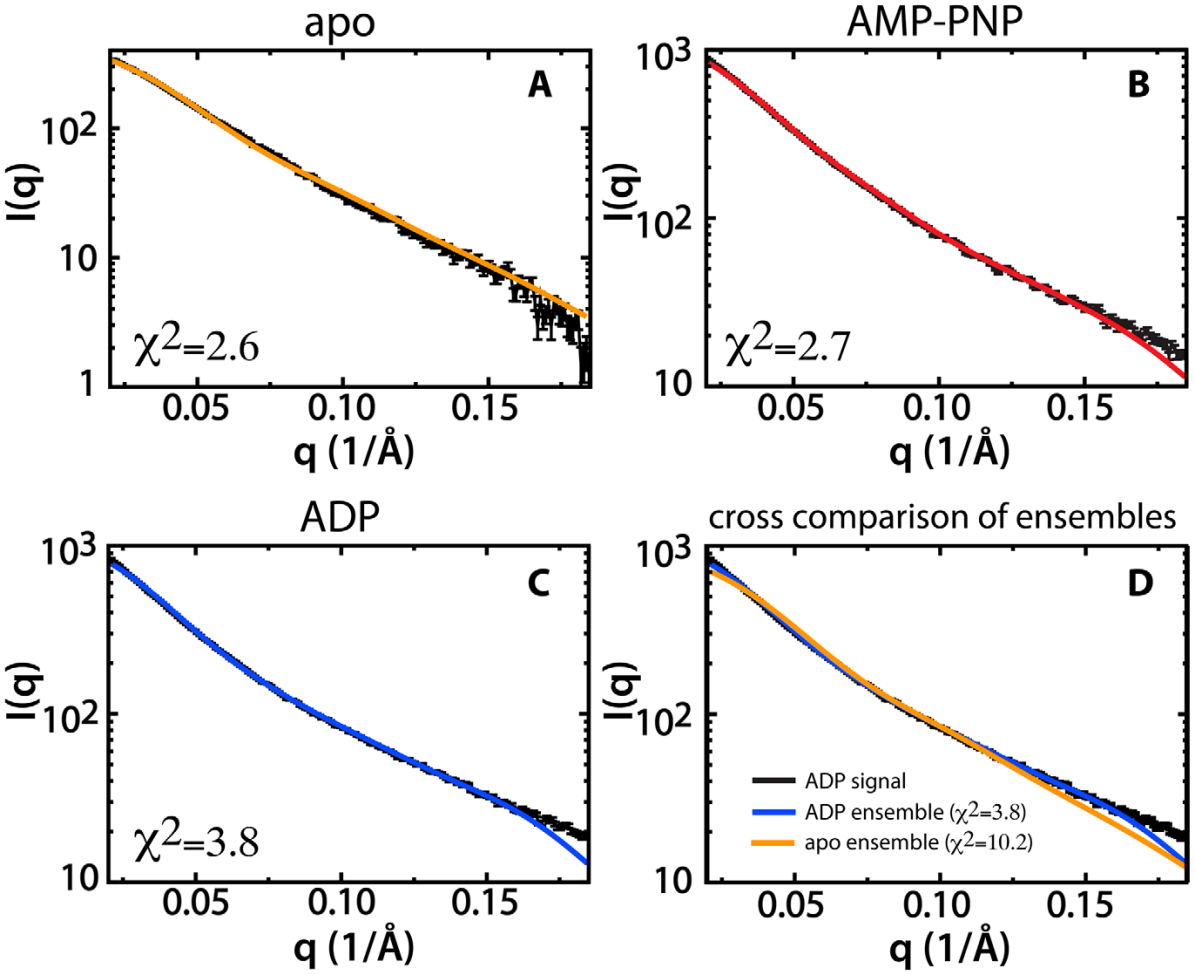
Theoretical scattering curves of the best-fitting conformational ensembles fitted to the experimental scattering curves (black) for apo Csk (A, orange), AMP-PNP bound Csk (B, red), and ADP-bound Csk (C, blue). The χ^2^ value for the ensemble fit to the experimental data appears in the lower left corner of the plot for each curve. A comparison of the fit of the apo Csk ensemble (orange) and the ADP-bound ensemble (blue) to the ADP experimental data (black) appears in D.

While the P(R_g_) analysis provides insight into the populations that are likely to be present in solution, it does not provide the probability of that population in solution. This is because each peak contains numerous conformations and possible bias by the simulation resulting in higher or lower sampling of a population. In order to quantify the probability of each population in solution we performed conformational ensemble analysis using conformations selected from the P(R_g_) analysis. We selected four representative conformations from the three most prominent P(R_g_) peaks for each state, where all the selected conformations are distinct (RMSD>2 Å). Representative structures selected for ensemble analysis appear in Figure 3. From this pool of candidate conformations we created all possible combinations that include one conformation from each population. Each combination was considered a candidate ensemble, with which we performed ensemble analysis (see Methods). For each weighted ensemble, we generated a theoretical scattering curve, which was then compared to the experimental data (Figure 4).

For each state of Csk, we find an ensemble containing conformations from the peaks in P(R_g_) that accounts well for the experimental data. The apo protein is heavily weighted towards compact conformations, where the best-fitting ensemble (χ^2^ = 2.6) arises from a mix of 90% peak 1 (∼34 Å) and 10% peak 3 (∼37 Å). The absence of peak 2 (∼35 Å) may be explained by a very small population or as a possible transient conformation between those represented at peaks 1 and 3. Both the AMP-PNP and ADP bound Csk conformational ensembles are comprised of extended structures. For the AMP-PNP bound protein, 40% of the best-fit ensemble is comprised of a conformation from the population of peak 1 (R_g_ ∼37 Å), with 30% each from peaks 2 (R_g_ ∼40 Å) and 3 (R_g_ ∼41 Å). This ensemble fits the experimental data with a χ^2^ = 2.7. When ADP is bound to Csk, there is a shift towards highly extended conformations with the best-fit ensemble consisting of 30% peak 1 (R_g_ ∼38 Å), 30% peak 2 (R_g_ ∼39 Å), and 40% peak 3 (R_g_ ∼43 Å), with χ^2^ = 3.8.

The population analysis results in a unique conformational ensemble for each state of Csk. The apo protein adopts primarily compact conformations, whereas the AMP-PNP bound protein adopts more extended conformations and the ADP bound protein adopts highly extended conformations. To confirm that each ensemble best describes that state of Csk, we fit the theoretical curves for all of the best-fit ensembles to the experimental data of all other states of Csk (Figure 4, Table 1). This comparison shows that the best-fit ensemble for each set of data describes the data better than any other ensemble does, reinforcing the notion that a unique solution is necessary for each state of Csk to describe the protein conformationally in solution.

**Table 1:** Cross-calculation of conformational ensembles.

Best-fit Conformational Ensemble from:	?^2^ calculated for experimental signal of:		
	Apo	AMP-PNP	ADP
Apo	2.6	6.4	10.2
AMP-PNP	5.1	2.7	4.6
ADP	6.0	3.2	3.8

The best-fit ensemble for each state of Csk was fit to the experimental data for all other states of Csk.

## Discussion

### Binding of Nucleotide Shifts the Conformational Ensemble towards Extended Conformations

Using SBMs to model SAXS data, we find Csk adopts a variety of conformations in solution that range from compact (R_g_ = 34 Å) to highly extended (R_g_ = 43 Å). For every state of Csk examined here, the scattering data are best described by an ensemble of conformations, not a single structure. Interestingly, the P(R_g_) distributions reveal a common population for the apo, AMP-PNP bound, and ADP bound protein, centered around 37 Å. For the apo protein, this population accounts for 10% of the best-fit ensemble and is the most extended conformation present in that ensemble. When AMP-PNP is bound to Csk, this population accounts for 40% of the best-fit ensemble, with the remainder of the ensemble composed of more extended conformations. Binding of ADP shifts this ensemble even further towards extended conformations, but still contains a contribution from the population common to all states (30%). Notably, 40% of the best-fit ensemble is comprised of a conformation from peak 3 (43 Å), a conformation that does not appear in the P(R_g_) for either the apo protein or the AMP-PNP bound protein.

This shift in the ensemble upon nucleotide binding, while surprising for a protein kinase, is consistent with observations for other proteins. Many recent studies highlight the role of dynamics and shifts in population during kinase catalysis. The energy landscape of PKA-C, as characterized using NMR and molecular dynamics, shows the apo enzyme can explore the landscape and access open and closed conformations, while ligand binding (nucleotide, substrate, or inhibitor) drives the enzyme to select alternate conformational states [30]. Combined crystallographic and NMR studies on arginine kinase display large substrate-induced domain motions and also show that the solution structure of the substrate-free protein is an equilibrium between substrate-bound and substrate-free forms [31]. The catalytic rates of two different forms of adenylate kinase are correlated with the timescales of motion in hinge and lid regions of the protein [32]. Similarly, allosteric effects upon ligand binding are observed in other nucleotide binding proteins. Solution NMR experiments on the nucleotide binding domain of the chaperone Hsp70 have revealed significant rotation in the subdomains when comparing the ATP and ADP bound states [33]. These changes impact the accessibility of a hydrophobic surface cleft distal from the nucleotide binding site, which is known to be essential for communication between the nucleotide binding domain and substrate binding domain. Allosteric effects are also observed upon cAMP binding to the exchange protein directly activated by cAMP (EPAC), where long-range communication between the phosphate binding cassette and the N-terminal helical bundle is transmitted via two intramolecular pathways [34]. Interconversion between competing conformations is also critical in the function of kinase adaptor proteins, such as Crk where proline isomerization switches the protein between an autoinhibitory conformation and an uninhibited, active conformation [35].

In the SAXS studies for Csk, the extended conformations of the nucleotide-bound states arise primarily from rearrangement of the regulatory domains. The observed extension of the SH2-kinase linker and SH3-SH2 linker may allow the domains to more easily rearrange to both make catalytically functional interdomain contacts and interact with other binding partners (discussed below). Communication between the SH2 and kinase domains is necessary for efficient catalysis as indicated by mutational studies of F183, a critical hydrophobic residue in the SH2-kinase linker [17]. When F183 is mutated to a glycine this communication is broken and catalytic efficiency decreases nearly 1000-fold. Interdomain communication between the SH3 and kinase domains also aids in catalysis. Mutations in the SH3-SH2 linker decrease catalytic activity up to five-fold by disrupting the interaction surface between the domains characterized by NMR [36].

### The Conformational Ensemble Provides Insight into Catalytic Cycling

Kinetic experiments using a short peptide substrate revealed that Csk exists in both low and high activity forms [15]. Overall, the high activity form represents only a small fraction of the total Csk, based on ‘burst’ amplitudes in pre-steady-state kinetic experiments. Interestingly, the present study also indicates that much of the enzyme in solution adopts open forms that are not expected to reflect high-activity states. In particular, the small and large kinase lobes adopt open rotomers in most calculated species, which are not expected to position ATP for highly productive phosphoryl transfer. In contrast, a higher population of Csk transiently adopts the active form when using Src rather than a peptide substrate in pre-steady-state kinetic experiments. This implies that a physiological target with increased enzyme-substrate contacts may promote a more closed, enzymatically-competent kinase domain. However, regardless of these substrate-dependent changes in conformer distribution detected in kinetic experiments, turnover is limited by a slow conformational change in Csk when using Src as a substrate [14]. The large changes observed between AMP-PNP- and ADP-bound forms in the SAXS-SBM study may correspond to the rate-limiting changes observed in the pre-steady-state kinetic experiments for Src phosphorylation. If the AMP-PNP- and ADP-bound Csk complexes reflect population distributions before and after Src phosphorylation, then the SAXS studies may effectively capture important forms of the enzyme within the catalytic cycle. Thus, the conversion of the more open forms observed in the ADP-bound state into the more closed forms in the ATP-bound complex may be correlated with the observed slow conformational changes in Csk kinetic experiments. These finding are consistent with recent work on Abl kinase, where the ADP-bound kinase domain is more open and exhibits increased flexibility [37].

### Csk Conformational Ensembles May Facilitate Signaling Processes

Csk is capable of interacting with a broad range of adaptor proteins through its SH2 domain [38]. These interactions likely require a high degree of adaptation and flexibility as Csk adopts a cytoplasmic location and then migrates to the plasma membrane in a phosphorylation-dependent manner [39]. This trafficking of Csk between the cytoplasm and membrane phospho-receptors may be facilitated by the regulatory SH2 and SH3 domains, which can transiently detach from the kinase core and become available for recognition at the plasma membrane. Diffusion of the SH2 domain is faster than the full protein, which may allow Csk to search for an SH2 domain binding partner more quickly. Such a scenario is reminiscent of the ‘fly-casting’ model for intrinsically disordered proteins, where unstructured regions of a protein may offer an increased capture radius for substrate recognition of specific targets [40]. Similarly, linker flexibility in Csk may allow the SH2 domain to effectively search for adaptors and then draw the kinase to the membrane for Src down-regulation. The SAXS-SBM results reported in this study provide new insights into these potential motions and how they may be coupled to both signaling and substrate processing in the cell.

## Methods

### Protein Expression and Purification

The full-length Csk protein was expressed in *Escherichia coli* strain BL21(DE3) [41], and purified by Ni^2+^ affinity chromatography [42]. The purified full-length enzyme was dialyzed against 50 mM Tris-HCl (pH 8.0), 150 mM NaCl, 5 mM DTT with 15% (v/v) glycerol and then concentrated to 107 µM and stored at −80°C.

### Small-angle X-ray Scattering Measurements

SAXS data were collected at 25°C at beamline 4-2 of the Stanford Synchrotron Radiation Laboratory. Scattering was independent of protein concentration (0.5 mg/mL–2.5 mg/mL) indicating that interparticle ordering and aggregation are negligible. The AMP-PNP bound samples were generated through addition of AMP-PNP in a 50∶1 molar ratio to protein as well as the addition of 10 mM MgCl_2_. Both AMP-PNP and MgCl_2_ were added from stocks prepared in Csk dialysis buffer. The ADP samples were prepared in the same manner. Data shown were obtained with 1 mg/mL protein for the apo and AMP-PNP bound proteins and 1.8 mg/mL for the ADP bound protein. A more detailed description of SAXS methodology for the study of proteins in solution may be found elsewhere [43].

### Small Angle Scattering Data Analysis

The data were converted from a TIFF image to I(q) versus *q* using SasTool (http://ssrl.slac.stanford.edu/~saxs/analysis/sastool.htm). *q* is the angular dependence of the scattering profile, which can be expressed as *q* = 4π(sinθ/λ), where θ is half the scattering angle and λ is the wavelength of scattered X-rays. The radius of gyration, R_g_, was determined by Guinier analysis. The goodness of the linear fit of the data in the low q range indicated no significant non-specific aggregation of the protein.

### Analysis of the Structural Ensemble via Molecular Dynamics Simulations

We employed molecular dynamics simulations with an all-atom structure based model to generate an ensemble of candidate conformations (i.e. structures that may explain the scattering profile). A detailed description of the all-atom model may be found elsewhere [44]. From this ensemble, theoretical scattering profiles were generated, and these profiles were compared to the experimentally measured data.

We used an all-atom structure-based forcefield for the individual domains but only include steric interactions between domains. These models are based on the concepts of energy landscape theory [45], [46], [47], have a low computational cost, and provide dynamic descriptions of proteins [48], [49], [50], [51] and assemblies [52] that are in good agreement with experiments. The functional form of the all-atom structure-based forcefield is
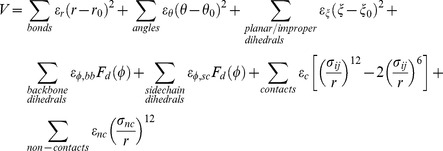
Where
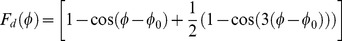

*r_0_, θ_0_, ξ_0_* and *φ_0_* are given the values found in the crystal structure (PDB code: 1K9A) [29] and σ = 2.5 Å, ε_r_ = 100/Å^2^, ε_θ_ = 20/rad^2^, ε_ξ_ = 10/rad^2^ and ε_nc_ = 0.01. Each native atom-atom contact interacts *via* a Leonard-Jones 12-6 interaction, where the energetic minimum corresponds to the native distance 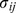 (as defined by the crystal structure). A contact is defined as any atom pair that is (a) separated by less than 6 Å, is (b) separated by at least 4 residues in sequence, and (c) has no atom between them (i.e. the “Shadow Algorithm”[53]). ε*_nc_* and σ*_nc_* define the excluded volume of each atom. Explicit representation of the atoms ensures that non-physical states, such as those with overlapping atoms or unrealistic bond angles, are disallowed. Contact and dihedral interactions were weighted as previously described [44]. In order to facilitate rapid sampling of all possible domain configurations, we removed all stabilizing inter-domain interactions and gave no configurational bias to the non-rigid dihedral angles (i.e. dihedrals not restrained by orbital hybridization) in the linkers.

The forcefield files for Gromacs [54] were generated by an online resource (http://smog.ucsd.edu) [55]. A timestep of 0.0005 time units was used and the simulation was coupled to a temperature bath *via* Langevin dynamics. The total simulation time was 20,000 time units, which corresponds to approximately 100 µs [56].

Similar to previous studies [57], [58], [59], we compare the candidate conformations from the simulation to the SAXS data by generating theoretical SAXS profiles for each conformation. Theoretical scattering curves were generated for all candidate conformations and ensembles using the CRYSOL software package [60]. Default parameters were used with the following exceptions: maximum order of harmonics = 50, order of Fibonacci grid = 18, number of points = 100, and maximum s-value = 0.18. Here, we measure the goodness of fit of each structure by calculating χ^2^, which is defined as,
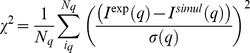
where *N_q_* is the number of data points in the scattering curve, *I*(*q*) is the SAXS intensity and σ(*q*) is the experimental error of *I^exp^*(*q*).

### Ensemble Analysis of the Candidate Conformations

Theoretical SAXS profiles were generated for linear combinations of conformations,
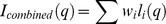
where *I_i_*(*q*) is the intensity profile of simulated conformation *i* and *w_i_* is the weight of conformation *i*. *I_combined_*(*q*) was determined for all combinations of *w_i_* = 0.1n, where n is an integer of value 0 to 10. χ^2^ was then calculated between each *I_combined_*(*q*) and the experimental profile to determine the set of *w_i_* values that minimize χ^2^.
